# Documentation Gaps in Suicide Risk Assessments: A Resident-Level Clinical Audit Using the C-SSRS Standard

**DOI:** 10.1192/bjo.2026.11569

**Published:** 2026-06-30

**Authors:** IQRA AIN ALI, Imtiaz Ahmad Dogar, Hafiza Aneeqa Mustafa, Abdul Rehman Asgher, Fatima Tuz Zahra

**Affiliations:** Faisalabad Medical University, Faisalabad, Pakistan

## Abstract

**Aims:**

Suicide remains one of the leading causes of death worldwide. It kills over 700,000 people every year. The most crucial steps to prevent suicide throughout the world are the accurate identification and good recording of suicidal risk. The Columbia-Suicide Severity Rating Scale (C-SSRS) is a validated tool that is commonly recommended in the evaluation of suicidal thoughts and behaviors, but clinical practitioners are experiencing difficulties in practicing it in an effective way, especially in emergency departments and psychiatric clinics. The psychiatry residents are typically the first to be consulted, and they have an instrumental role in conducting and recording the suicide risk assessment. This clinical audit was to evaluate how properly the psychiatry residents documented their suicide risk assessments using the C-SSRS as a standard.

**Methods:**

A retrospective clinical audit at the department of psychiatry was organized. The researchers examined the psychiatric records of adult patients seen by residents during a three-month period. A standardized audit checklist based on C-SSRS criteria was used to evaluate the documentation of suicidal ideation, suicidal behavior, intensity and frequency of thoughts, use of C-SSRS, risk stratification, protective factors, and safety planning. The reporting of ideation and behavior and the use of a structured tool were expected to be 100% according to compliance standards, while risk level and safety planning were expected to be 90%.

**Results:**

A total of 118 patient charts were analyzed. Most of the notes (79.7%) indicated that the patients entertained suicidal thoughts, and only 47.5% of the notes indicated that the patients attempted suicidal behavior. Only a paltry 25.4% of the hospital records included the extent or frequency of the suicidal thoughts. The application of the Columbia-Suicide Severity Rating Scale was only documented in one case, equal to 0.8%. Almost all of the cases never provided risk level, protective factors, and safety plans, standing at 2.5%, 0.8%, and 0.8%, respectively. The lack of documentation was consistent throughout all residency training levels, and it was not an experience-based problem.

**Conclusion:**

The audit found that the medical records of the patients were not adequate. A validated tool to evaluate suicide death risk was not utilized. There is an urgent need to train residents, adjust the suicide evaluation, and introduce the ideas of C-SSRS into electronic records and review them regularly. Recording proper notes is a vital aspect of suicide prevention that keeps patients safe. It allows hospitals and staff to provide more effective care.

